# Perspectives of at-Risk Individuals on Preventive Intervention for Rheumatoid Arthritis: A Mini Review

**DOI:** 10.3389/fimmu.2022.883287

**Published:** 2022-04-29

**Authors:** Marie Falahee, Karim Raza

**Affiliations:** ^1^ Rheumatology Research Group, Institute of Inflammation and Ageing, College of Medical and Dental Sciences, University of Birmingham, Birmingham, United Kingdom; ^2^ Medical Research Council (MRC) Versus Arthritis Centre for Musculoskeletal Ageing Research and the Research into Inflammatory Arthritis Centre Versus Arthritis, University of Birmingham, Birmingham, United Kingdom; ^3^ Rheumatology Department, Sandwell and West Birmingham National Health Service (NHS) Trust, Birmingham, United Kingdom; ^4^ National Institute for Health and Care Research (NIHR) Birmingham Biomedical Research Centre, University Hospitals Birmingham National Health Service (NHS) Foundation Trust and University of Birmingham, Birmingham, United Kingdom

**Keywords:** rheumatoid arthritis, prediction, prevention, at-risk groups, perceptions, preferences, choice - behaviour

## Abstract

There has been intense research focus on the biological mechanisms underlying the transition from health to disease for rheumatoid arthritis (RA) over recent years, and it is now well established that a state of autoimmunity precedes the development of symptoms for a large proportion of patients. This has led to an increased interest in the identification of at-risk groups and the potential for preventive intervention. The ability of several immunomodulatory agents to delay or prevent RA is under investigation and novel cellular therapies are in development. Preventive approaches are also being assessed in other chronic autoimmune diseases. For example, an anti-CD3 antibody has recently been shown to delay progression to type 1 diabetes in non-diabetic relatives of patients identified as being at high risk. The identification and treatment of individuals as being at risk of a disease where there is a degree of uncertainty around the potential for benefit is socially and ethically challenging. Recently reported difficulties in recruitment to RA prevention trials have underlined the importance of understanding the perspectives of at-risk individuals to identify barriers and facilitators that need to be addressed in order for preventive strategies to be acceptable. Understanding of their preferences for benefits and risks of preventive interventions can inform efficient intervention prioritization, prevention trial design and the development of informational resources for those at risk. In this review we summarize current knowledge of preferences for RA prevention and make recommendations for further research needed to ensure efficient development of preventive therapies and clinical implementation.

## Introduction

Rheumatoid arthritis (RA) is an inflammatory disease that causes painful swelling of the joints, fatigue, depression, and extra-articular manifestations including accelerated cardiovascular disease. There is currently no cure, and long-term treatment is usually required to prevent joint erosion and loss of function (1). Although the introduction of biologic and targeted synthetic disease modifying anti-rheumatic drugs (b/ts DMARDs) has revolutionized management of RA, approximately 10-15% of patients do not respond to multiple sequential therapies (2). Risks of treatments for RA include infection and lung, liver and haematological toxicity. In addition to the disease burden experienced by patients, RA presents a significant socioeconomic burden (3, 4). There is thus a clear rationale for the development of a cure and/or preventive interventions for this condition.

It is established that early treatment of RA is associated with improved outcomes (5). This has led to increased focus on the earliest stages of disease development, including pre-clinical phases (6). Understanding of the biological mechanisms operating at articular and extra-articular sites in at-risk individuals has evolved rapidly (7), and algorithms to predict the development of clinical arthritis in at-risk populations have become increasingly sophisticated (8). Recognition of groups at risk of RA presents possibilities for preventive intervention. Such intervention could prevent or delay the onset of clinical arthritis, and also reduce the complex symptom burden often experienced before diagnosis (9). Intervention at this stage could also reduce RA severity if it were to subsequently develop.

The European Alliance of Associations for Rheumatology (EULAR) has provided recommendations for terminology to identify distinct at-risk phases (based on genetic and environmental risk factors, RA-related autoantibodies and symptoms) (10). Key target groups for preventive approaches may have one or more of the following: (a) genetic risk factors (e.g. risk is increased approximately fourfold in first-degree relatives [FDRs) (11)]; (b) environmental risk factors [e.g. smoking (12)]; (c) systemic autoimmunity associated with RA (typically indicated by rheumatoid factor and/or anti–citrullinated protein/peptide antibodies); (d) symptoms suggestive of underlying inflammation but without clinically apparent synovitis [clinically suspect arthralgias (CSAs) (13)]; or (e) early arthritis that does not fulfil RA classification criteria. Different approaches are likely to be appropriate at each phase. Primary prevention of seropositive RA would involve intervention to prevent development of systemic autoimmunity, while secondary prevention of seropositive RA would involve prevention of RA development in individuals with pre-existing systemic autoimmunity (6).

EULAR guidance for trials and observational studies in individuals at-risk of RA, based on expert consensus and evidence from systematic reviews (14, 15), is now available and the scene is set for progress towards a new paradigm of prevention, rather than treatment of RA (16). Evaluation of candidate preventive therapies for RA is a nascent research area, though early findings are promising. Whilst intramuscular glucocorticoid did not delay arthritis development in seropositive arthralgia patients (17), it prevented 10% of patients with early inflammatory polyarthritis from progressing to RA and delayed DMARD prescription (18). B-cell depletion with a single infusion of rituximab delayed, but did not prevent RA onset in individuals with seropositive arthralgia and either imaging synovitis or evidence of an acute phase response (19). The effects of time-limited courses of other immunomodulatory therapies, including abatacept (20) and hydroxychloroquine (21), on RA development are currently being assessed in other at-risk groups, including asymptomatic FDRs (21). Preventive treatments are also under investigation in other chronic autoimmune conditions. For example, an anti-CD3 antibody delayed progression to type 1 diabetes in non-diabetic relatives of patients identified as being at high risk based on the presence of diabetes-related autoantibodies and other risk factors (22).

Although trials of lifestyle interventions to prevent RA are currently lacking, Vitamin D supplementation for five years has been shown to reduce risk of autoimmune diseases (23). Omega 3 fatty acids have been inversely associated with the presence of RA-related autoantibodies (24, 25), though a prospective cohort study did not find an association between fish intake with RA development (26). There is a robust rationale for studies of smoking cessation to reduce risk of RA (12, 27, 28), and other interventions such as periodontal treatment and weight control have preventive potential (29, 30).

Whilst prevention of diseases such as RA has considerable potential to improve outcomes and reduce societal costs, the identification of individuals as being at risk, and the use of preventive treatment where there is a degree of uncertainty around disease development and progression, is ethically challenging (31, 32). Those at risk may face complex decisions around accepting predictive assessments and risks associated with immunomodulatory interventions in exchange for uncertain benefit. A recent trial of 40mg atorvastatin daily for three years to prevent arthritis development in seropositive arthralgia patients was terminated prematurely due to unwillingness to participate (33). A related qualitative study exploring barriers to trial participation highlighted perceptions that the need for treatment was low and outweighed by concerns about treatment risks and the burden of trial participation (34).

Understanding the perceptions and preferences of those at risk for preventive approaches is therefore essential to inform the development of balanced, tailored informational resources for those considering trial participation, and to support efficient clinical translation. There is increasing recognition of the value of information about patient preferences for decision-making by the pharmaceutical industry, regulatory agencies, and health technology assessors (35–37). Systematically collected data on patient preferences can support efficient, patient-focused medicine development, including target product profile development, endpoint selection, benefit-risk assessment, and regulatory approval (38, 39). The integration of patient preference information into drug development is more likely to result in treatments that are acceptable to patients. This is especially important in the context of disease prevention, where uptake and adherence to medications can be low (40, 41). Therefore, the objective of this article is to provide a narrative review of what is known about the perceptions and preferences of at-risk populations (EULAR at-risk stages a-d) and other key stakeholders for predictive and preventive strategies for RA, and identify opportunities for further investigation. The search strategy used to identify relevant literature is summarized in **Supplementary Material**.

## Exploratory Qualitative Studies

A summary of published qualitative investigations exploring perceptions of predictive testing and/or preventive interventions for RA can be found in **Table 1**. Perceptions of predictive approaches have been studied in those with CSA (43, 48), asymptomatic individuals who have tested positive for RA-related autoantibodies (48), FDRs (44), the general public (49), and RA patients (who may be involved in providing access and/or information to FDRs) (45). Participants across these studies recognized the value of disease risk information in terms of increased self-awareness and also the potential for early or preventive treatment (15). However, several studies noted concerns around the uncertainty associated with disease development and potential for psychological distress (44, 45, 48). Mosor et al. (2020) reported that these concerns were particularly salient for participants with joint symptoms (48). However in another study, FDRs who received personalized risk education reported greater levels of reassurance than those who received standard RA risk information (50).

**Table 1:** Summary of published qualitative studies exploring perceptions/preferences of RA prediction/prevention.

Participants	Study Objectives	Methods	Key findings	Authors
20 FDRs* taking part in an observational cohort study (Switzerland)	Explore perceptions of preventive treatments and participation in interventional trials to prevent RA	Interviews	Preventive treatments with low risk of serious adverse effects were acceptable when risk of RA was above 30%.	Novotny et al, (42)
4 CSA** patients taking part in an observational cohort study (Netherlands)	Explore perceptions of CSA and prognostic information about RA	Focus Groups	Negative views about numerical risk estimates	Newsum et al, (43)
32 FDRs recruited *via* RA patients (UK, Germany, Austria)	Explore perceptions of RA risk and predictive testing	Interviews	Unmet information needs and concerns about uncertainty/anxiety	Stack et al, (44)
22 RA patients (UK)	Explore perceptions of predictive testing, preventive intervention and communicating with relatives about RA risk	Interviews	Positive views associated with misperceptions about risk information. Selective family communication about risk	Falahee et al, (45)
32 FDRs recruited *via* RA patients in secondary care clinics (UK, Germany, Austria)	Explore perceptions of preventive interventions for RA	Interviews	Lifestyle interventions preferred. Drugs appropriate after symptom onset. Concerns about drug side effects.	Simons et al, (46)
25 participants (13 patients, 5 FDRs and 7 rheumatologists	Define attributes of treatments to prevent RA to be assessed in a quantitative study	Focus groups	Role of healthcare professional recommendation in treatment decisions highlighted	Munro et al, (47)
34 seropositive individuals (24 CSA patients and 10 asymptomatic individuals attending extended health examination) (Austria, Germany, UK)	Explore perspectives and information needs around predictive test results and preventive treatment	Interviews	Symptomatic individuals more likely to accept preventive intervention and experience anxiety	Mosor et al, (48)
18 seropositive CSA patients invited to take part in interventional trial to prevent RA development (Netherlands)	Identify barriers and facilitators to participation in trial to prevent RA development	Focus groups	Identified information needs of trial participants highlighting potential for benefit and addressing concerns about burden of trial participation	Van Boheemen et al, (34)
21 members of the public (UK)	Perceptions of predictive testing for RA, breast cancer and early onset Alzheimer’s disease	Focus groups	Concerns around predictive testing less pronounced for RA. RA perceived to be less serious than other diseases.	Singhal et al, (49)

*FDR, First-degree relative; **CSA, Clinically suspect arthralgia.

In a focus group study of CSA patients, participants had negative views of the utility of numerical information about risk (43). Interview studies with FDRs (44) and patients (45) suggested that positive views of predictive testing for RA were associated with the misperception that such tests could rule in/out RA. Negative viewpoints were associated with an understanding of the probabilistic nature of risk information (45). In focus groups, members of the general public reflected misperceptions about the severity of RA that had been found in previous studies, and held beliefs that risk assessment was more appropriate for diseases that were perceived to be more serious (49). Lack of public awareness about the negative personal impact of RA was highlighted by RA patients as a potential barrier to predictive strategies (45). Several studies emphasized unmet needs for information about RA and risk factors for RA (44, 45, 48).

The first qualitative study addressing perspectives on preventive treatments for RA found that most participants would accept a prophylactic treatment if their risk of developing RA was 30% or greater (42). However, the participants in that study were FDRs enrolled in a prospective observational cohort and their views may not be representative of other at-risk groups. Other studies of FDRs and RA patients (45–47) suggested that lifestyle interventions would be preferred over pharmaceutical therapies, highlighting concerns about medication side effects and beliefs that drug treatment is appropriate only after symptoms have developed. Such beliefs were echoed by Mosor et al. (2020) who reported that seropositive individuals without symptoms were less inclined to consider preventive treatments than those who were experiencing arthralgia (48). The focus group study by Munro et al. (2020) involving participants who were either RA patients, FDRs or rheumatologists also found that the precision of disease risk estimates and endorsement by a trusted healthcare professional would be important considerations when deciding whether to accept a preventive treatment for RA (47). No other qualitative studies published to date have addressed the perspectives of healthcare professionals.

Many of the themes described above were also found in interviews with autoantibody positive individuals with CSA who had been invited to take part in a trial of a treatment to reduce their risk of developing RA (34). Whilst potential for personal and societal benefit, along with detailed information and support from the individual’s physician, facilitated trial participation, barriers included beliefs about personal risk status and the need for treatment, and concerns about treatment-related harms and the perceived burden of trial participation.

## Quantitative Investigations

**Table 2** summarizes published quantitative investigations. A survey study found that over 50% of FDRs were definitely interested in taking a predictive test to quantify their risk of developing RA (52). Predictors of levels of interest included attitudes about risk knowledge, information-seeking preferences and beliefs that predictive testing could cause psychological harm. No other quantitative studies have addressed preferences for predictive testing for RA.

**Table 2:** Summary of published quantitative studies assessing perceptions/preferences of RA prediction/prevention.

Participants	Study Objectives	Methods	Key findings	Authors
32 FDRs* taking part in an observational cohort study (Switzerland)	Assess impact of treatment efficacy, mode of administration, severe side effects and mild side effects on likelihood of acceptance of preventive treatment for RA	Stated choice survey (best-worst scaling)^1^	Hypothetical RA risk status affected likelihood that treatment chosen. Treatment effectiveness and severe side effects significantly affected choices, mild side effects and mode of administration did not.	Finckh et al, (51)
288 self-reported FDRs recruited *via* Amazon’s Mechanical Turk platform	Assess relative importance of, and trade-offs between, preventive treatment effectiveness, side effects, mode of administration, certainty in evidence for effectiveness, and healthcare professional endorsement	Stated choice survey (discrete choice experiment) ^2^	Method of administration, effectiveness, healthcare professional endorsement and serious side effects were most influential determinants of choices. Predicted uptake of biological therapies was low.	Harrison et al, (52)
108 participants (78 RA patients, 30 of their FDRs and 39 rheumatologists) (Canada)	Assess relative importance of, and trade-offs between, preventive treatment effectiveness, side effects, mode of administration, certainty in evidence for effectiveness, and rheumatologist/patient endorsement	Stated choice survey (discrete choice experiment) ^2^	Rheumatologist/patient endorsement most important attribute. Non-biologic therapies preferred. Preferences of patients and FDRs differed from those of rheumatologists	Harrison et al, (53)
187 participants (100 seropositive CSA** patients, 38 FDRs of patients with axial spondylitis, 49 rheumatologists) (Netherlands)	Assess willingness to accept 100% effective preventive treatments with no/minor side effects at 30%/70% disease risk	Survey	Lifestyle interventions were acceptable to participants, but rarely discussed by rheumatologists. Acceptability of drug treatment was higher amongst rheumatologists than at risk individuals. Treatment acceptability increased with hypothetical risk of RA	Van Boheemen et al, (54)
396 FDRs of RA patients (UK)	Assess interest in taking a predictive test for RA, and predictors of interest.	Survey	FDRs interest in predictive testing was high. Predictors of interest included information-seeking preferences and beliefs that predictive testing would increase empowerment or cause anxiety	Wells et al, (55)

*FDR, First-degree relative; **CSA, Clinically suspect arthralgia.

^1^Stated choice study design informed by Novotny et al. (2013) qualitative study (42).

^2^Stated choice study design informed by Munro et al. (2018) qualitative study (47).

Van Boheemen et al. (2020) surveyed willingness to use 100% effective preventive medications amongst seropositive arthralgia patients and rheumatologists (54). At 30% baseline risk of developing RA, 53% of patients and 74% of rheumatologists would be willing to use a preventive therapy with no side effects. At 70% baseline risk, this increased to 69% for patients and 92% for rheumatologists. A drug with minor side effects was acceptable to 26% of patients and 31% of rheumatologists when the baseline risk of RA was 30%; and to 40% of patients and 76% of rheumatologists when risk of RA was 70%. Patients’ willingness to make preventive lifestyle changes was high, though this was not often the focus of rheumatologists’ consultations (54).

Stated choice methods, where participants choose between hypothetical treatment options described by treatment attributes (e.g., risks, benefits, method of administration, etc.) with pre-specified levels that are varied systematically, provide quantitative information about the relative importance of treatment attributes, benefit/risk tradeoffs, preference heterogeneity, and predicted uptake. Such information can inform selection of outcomes and endpoints in clinical trials and also support stakeholder (e.g. regulator, HTA) decision-making (38, 39). Whilst stated preferences for RA treatments have been widely assessed (56) there are limited examples for RA prevention (57).

A best-worst scaling study of 32 FDRs enrolled in a prospective cohort in Switzerland reported that treatment effectiveness to reduce risk of RA and the likelihood of serious adverse effects were significant determinants of the likelihood that participants would choose a preventive treatment (51). Mild adverse events and the method of drug administration did not influence participants’ decisions. Preventive therapies were chosen 7%, 30% and 38% of the time when participants assumed a baseline risk status of 1%, 20%, and 40%, respectively (51).

A larger sample of self-reported FDRs took part in a Canadian discrete choice experiment (DCE) (52). Participants were asked to assume a 60% risk of developing RA. Method of administration, treatment effectiveness, healthcare professional preference and risk of serious side effects were the treatment attributes that most influenced participants’ choices. Latent class analysis identified three sub-groups of participants whose preferences were driven not only by treatment effectiveness, but also by safety aspects, healthcare professional endorsement and treatment convenience, respectively. Predicted uptake was high for non-biologic drugs such as hydroxychloroquine (84%), but low for atorvastatin and biologics (52).

Nonbiologic drugs were also preferred in a similar survey in Canada of a sample including RA patients, FDRs and rheumatologists (53). 38% of patients/FDRs preferred no preventive treatment, compared with 12% of rheumatologists. The most important drivers of participants’ choice were shared decision-making (whether the treatment option was supported by the rheumatologist/patient), risks of serious side effects, and treatment effectiveness (53).

Finally, the protocol of a stated choice survey employing both a DCE and a probabilistic threshold technique to assess preferences for preventive treatments for RA has been published (58). That study recruits large samples of the general population *via* survey panels in the UK, Germany and Romania, and also recruits FDRs of confirmed RA patients. Initial findings from the DCE of the general population indicated that treatment effectiveness was the most important determinant of choice across countries, and the sample in Romania was more sensitive to treatment risks (59). Predicted uptake of profiles resembling RA prevention candidate therapies varied across countries, with a profile chosen to estimate abatacept being most likely treatment to be chosen in all three (59).

## Discussion

The studies described in this narrative review highlight significant progress in our understanding of preferences for risk assessment and preventive interventions for RA (60). There are now a number of qualitative explorations across a range of stakeholder groups indicating perceived potential for benefit that is sometimes outweighed by concerns around the probability of RA development, treatment harms, uncertainty about effectiveness, and perceptions that preventive intervention with pharmaceutical products are not warranted for RA. The latter finding may reflect commonly held public misperceptions that RA is not a serious condition, and/or that it is a natural part of human ageing (61–63). Taken together these studies highlight an urgent need to provide at risk groups with accurate information about RA, RA risk and the risks and benefits associated with potential preventive strategies to support shared decision-making in the context of trial participation and effective clinical translation. Little is known about the perspectives of healthcare professionals in this context. As the implementation of preventive strategies for RA would require considerable reconfiguration of healthcare services, further studies are needed.

Whilst several studies have described a preference for lifestyle interventions over pharmaceutical therapies, and personalized risk education has been shown to increase risk-reducing health behaviours amongst FDRs of RA patients (64), interventional trials of potential preventive lifestyle interventions for RA (such as smoking cessation, periodontal treatment, weight loss and dietary change) are currently lacking.

There are fewer examples of quantitative studies. Choice-based methods have been applied to samples of FDRs and the public and provide initial evidence that preventive treatments for RA are acceptable to those assuming a hypothetical high-risk status. However, no quantitative studies have used stated choice methods to directly elicit the preferences of very high-risk populations (e.g., seropositive individuals with CSA) for either predictive tools or preventive treatments. Further research in this area is therefore needed to enable quantification of the relative importance of outcomes/intervention attributes, benefit/risk tradeoffs and predicted uptake of treatment profiles for this group. Such information would support patient-focused development of preventive therapies and enhance the likelihood of clinical impact. Importantly, no stated choice studies have quantified the degree of benefit required from preventive lifestyle interventions for RA in exchange for sustained behavioral change. This, is an important area for future research given that several studies have indicated that lifestyle interventions are preferred for prevention of RA. No studies to date have assessed preferences for combined lifestyle and pharmacological intervention.

All preference studies undertaken to date have focused on a single aspect of treatment effectiveness: reduction of the risk of RA development. None have investigated preferences for outcomes such as delay of the onset of RA, or reduction of subsequent RA severity. For symptomatic at-risk groups, important additional benefits may include reduction of symptoms such as arthralgia and fatigue. Further research is therefore needed to quantify the relative importance of these outcomes in high-risk populations. All existing studies were undertaken in Europe or North America. Further investigation is needed to assess preferences in different countries with different types of healthcare provision and also in low and middle income countries. Existing choice-based studies have not yet identified participant characteristics (e.g., gender; health literacy; and numeracy) associated with preference heterogeneity (52), though this is currently under investigation (58).

Comparisons across quantitative studies are limited by methodological heterogeneity. For example, where a treatment attribute describing healthcare professional endorsement or certainty of risk estimates is included in the experimental design it is likely to be an important determinant of participants choices (52, 53). Such considerations can be held constant in the treatment scenario to allow assessment of the relative importance of additional treatment characteristics.

The emergence of evidence-based recommendations to guide the use of preference studies for decision-making in the medical product lifecycle, such as those produced by the PREFER consortium (35), provides a framework for future studies in this area. PREFER has also contributed to an agenda for further refinement of stated preference study methodology. For example, the application of measures of psychological constructs to explain preference heterogeneity (65, 66), and the development of scenario-based interactive educational tools to deliver background information and training to preference study participants to support informed choices (67). These methodological considerations are particularly relevant in the context of RA prevention, where decision making by those at risk of developing RA about accepting treatment is likely to be highly preference sensitive, and influenced by underlying beliefs about RA, personal risk status and treatment risks and benefits. Therefore, the development of innovative educational tools to obtain informed preferences within preference elicitation studies of preventive interventions for RA could also be usefully applied to support shared decision-making in clinical settings.

Preventive strategies for other chronic conditions are routinely integrated into clinical practice, and many asymptomatic individuals accept preventive pharmaceutical treatments (e.g., statins and antihypertensive medications are widely prescribed to reduce risk of cardiovascular disease). A similar approach to RA could dramatically improve clinical outcomes with considerable cost savings. The development of treatments to achieve this that are acceptable to those at risk would represent an important paradigm shift. Such an achievement is more likely to be realized if it is informed by an understanding of stakeholder perspectives and underpinned by evidence that aligns with the treatment preferences of at-risk populations.

## Author Contributions

Both authors contributed to the conception, writing and finalization of this article. MF wrote an initial draft. All authors contributed to the article and approved the submitted version.

## Funding

There was no specific funding for this work. KR is supported by the NIHR Birmingham Biomedical Research Centre.

## Conflict of Interest

KR declares personal fees from Abbvie, Sanofi, and grant/research support from Bristol Myers Squibb.

The remaining author declares that the research was conducted in the absence of any commercial or financial relationships that could be construed as a potential conflict of interest.

## Publisher’s Note

All claims expressed in this article are solely those of the authors and do not necessarily represent those of their affiliated organizations, or those of the publisher, the editors and the reviewers. Any product that may be evaluated in this article, or claim that may be made by its manufacturer, is not guaranteed or endorsed by the publisher.

## References

[1] Smolen JS Landewé RBM Bijlsma JWJ Burmester GR Dougados M Kerschbaumer A. EULAR Recommendations for the Management of Rheumatoid Arthritis With Synthetic and Biological Disease-Modifying Antirheumatic Drugs: 2019 Update. Ann Rheum Dis (2020) 79(6):685–99. doi: 10.1136/annrheumdis-2019-216655 31969328

[2] Nagy G Roodenrijs NMT Welsing PM Kedves M Hamar A van der Goes MC. EULAR Definition of Difficult-to-Treat Rheumatoid Arthritis. Ann Rheum Dis (2021) 80(1):31–5. doi: 10.1136/annrheumdis-2020-217344 PMC7788062 33004335

[3] National Rheumatoid Arthritis Society and University of Chester. The Burden of Rheumatoid Arthritis Across Europe: A Socioeconomic Survey (BRASS) - Summary Report. Available at: https://www.nras.org.uk/data/files/Publications/Surveys%20Reports/UoC_HCD_BRASS%20Summary%20Report%20FINAL.pdf.

[4] Hsieh P-H Wu O Geue C McIntosh E McInnes IB Siebert S. Economic Burden of Rheumatoid Arthritis: A Systematic Review of Literature in Biologic Era. Ann Rheum Dis (2020) 79:771–7. doi: 10.1136/annrheumdis-2019-216243 32245893

[5] van der Linden MP le Cessie S Raza K van der Woude D Knevel R Huizinga TW. Long-Term Impact of Delay in Assessment of Patients With Early Arthritis. Arthritis Rheumatol (2010) 62(12):3537–46. doi: 10.1002/art.27692 20722031

[6] Raza K Klareskog L Holers VM. Predicting and Preventing the Development of Rheumatoid Arthritis. Rheumatology (2016) 55(1):1–3. doi: 10.1093/rheumatology/kev261 26224307 PMC5854029

[7] Tracy A Buckley CD Raza K. Pre-Symptomatic Autoimmunity in Rheumatoid Arthritis: When Does the Disease Start? Semin Immunopathol (2017) 39(4):423–35. doi: 10.1007/s00281-017-0620-6 PMC5486797 28337522

[8] van Boheemen L van Schaardenburg D. Predicting Rheumatoid Arthritis in At-Risk Individuals. Clin Ther (2019) 41(7):1286–98. doi: 10.1016/j.clinthera.2019.04.017 31196647

[9] Stack RJ van Tuyl LHD Sloots M van de Stadt LA Hoogland W Maat B. Symptom Complexes in Patients With Seropositive Arthralgia and in Patients Newly Diagnosed With Rheumatoid Arthritis: A Qualitative Exploration of Symptom Development. Rheumatology (2014) 53(9):1646–53. doi: 10.1093/rheumatology/keu159 24729397

[10] Gerlag DM Raza K van Baarsen LGM Brouwer E Buckley CD Burmester GR. EULAR Recommendations for Terminology and Research in Individuals at Risk of Rheumatoid Arthritis: Report From the Study Group for Risk Factors for Rheumatoid Arthritis. Ann Rheum Dis (2012) 71(5):638–41. doi: 10.1136/annrheumdis-2011-200990 PMC3329228 22387728

[11] Frisell T Holmqvist M Källberg H Klareskog L Alfredsson L Askling J. Familial Risks and Heritability of Rheumatoid Arthritis: Role of Rheumatoid Factor/Anti–Citrullinated Protein Antibody Status, Number and Type of Affected Relatives, Sex, and Age. Arthritis Rheumatism (2013) 65(11):2773–82. doi: 10.1002/art.38097 23897126

[12] Kallberg H Ding B Padyukov L Bengtsson C Ronnelid J Klareskog L. Smoking is a Major Preventable Risk Factor for Rheumatoid Arthritis: Estimations of Risks After Various Exposures to Cigarette Smoke. Ann Rheum Dis (2011) 70(3):508–11. doi: 10.1136/ard.2009.120899 PMC3033966 21149499

[13] van Steenbergen HW Aletaha D Beaart-van de Voorde LJJ Brouwer E Codreanu C Combe B. EULAR Definition of Arthralgia Suspicious for Progression to Rheumatoid Arthritis. Ann Rheum Dis (2017) 76:491–6. doi: 10.1136/annrheumdis-2016-209846 27991858

[14] Mankia K Siddle H Di Matteo A Alpízar-Rodríguez D Kerry J Kerschbaumer A. A Core Set of Risk Factors in Individuals at Risk of Rheumatoid Arthritis: A Systematic Literature Review Informing the EULAR Points to Consider for Conducting Clinical Trials and Observational Studies in Individuals at Risk of Rheumatoid Arthritis. RMD Open (2021) 7(3). doi: 10.1136/rmdopen-2021-001768 PMC8449955 34531306

[15] Siddle HJ Chapman LS Mankia K Zăbălan C Kouloumas M Raza K. Perceptions and Experiences of Individuals at-Risk of Rheumatoid Arthritis (RA) Knowing About Their Risk of Developing RA and Being Offered Preventive Treatment: Systematic Review and Thematic Synthesis of Qualitative Studies. Ann Rheum Dis (2022) 81(2):159. doi: 10.1136/annrheumdis-2021-221160 34750103 PMC8762008

[16] Mankia K Siddle HJ Kerschbaumer A Alpizar Rodriguez D Catrina AI Cañete JD. EULAR Points to Consider for Conducting Clinical Trials and Observational Studies in Individuals at Risk of Rheumatoid Arthritis. Ann Rheum Dis (2021) 80(10):1286–98. doi: 10.1136/annrheumdis-2021-220884 PMC8458095 34362746

[17] Bos WH Dijkmans BAC Boers M van de Stadt RJ van Schaardenburg D. Effect of Dexamethasone on Autoantibody Levels and Arthritis Development in Patients With Arthralgia: A Randomised Trial. Ann Rheum Dis (2010) 69:571–4. doi: 10.1136/ard.2008.105767 19363022

[18] Verstappen SMM McCoy MJ Roberts C Dale NE Hassell AB Symmons DPM. Beneficial Effects of a 3-Week Course of Intramuscular Glucocorticoid Injections in Patients With Very Early Inflammatory Polyarthritis: Results of the STIVEA Trial. Ann Rheum Dis (2010) 69:503–9. doi: 10.1136/ard.2009.119149 19825849

[19] Gerlag DM Safy M Maijer KI Tang MW Tas SW Starmans-Kool MJF. Effects of B-Cell Directed Therapy on the Preclinical Stage of Rheumatoid Arthritis: The PRAIRI Study. Ann Rheum Dis (2019) 78:179–85. doi: 10.1136/annrheumdis-2017-212763 PMC6352407 30504445

[20] Al-Laith M Jasenecova M Abraham S Bosworth A Bruce IN Buckley CD. Arthritis Prevention in the Pre-Clinical Phase of RA With Abatacept (the APIPPRA Study): A Multi-Centre, Randomised, Double-Blind, Parallel-Group, Placebo-Controlled Clinical Trial Protocol. Trials (2019) 20(1):429. doi: 10.1186/s13063-019-3403-7 31307535 PMC6633323

[21] Strategy to Prevent the Onset of Clinically-Apparent Rheumatoid Arthritis (StopRA) . Available at: https://clinicaltrials.gov/ct2/show/NCT02603146.

[22] Herold KC Bundy BN Long SA Bluestone JA DiMeglio LA Dufort MJ. An Anti-CD3 Antibody, Teplizumab, in Relatives at Risk for Type 1 Diabetes. New Engl J Med (2019) 381(7):603–13. doi: 10.1056/NEJMoa1902226 PMC6776880 31180194

[23] Hahn J Cook NR Alexander EK Friedman S Walter J Bubes V. Vitamin D and Marine Omega 3 Fatty Acid Supplementation and Incident Autoimmune Disease: VITAL Randomized Controlled Trial. BMJ (2022) 376. doi: 10.1136/bmj-2021-066452 PMC8791065 35082139

[24] Gan RW Young KA Zerbe GO Demoruelle MK Weisman MH Buckner JH. Lower Omega-3 Fatty Acids are Associated With the Presence of Anti-Cyclic Citrullinated Peptide Autoantibodies in a Population at Risk for Future Rheumatoid Arthritis: A Nested Case-Control Study. Rheumatol (Oxford England) (2016) 55(2):367–76. doi: 10.1093/rheumatology/kev266 PMC5009416 26370400

[25] Gan RW Demoruelle MK Deane KD Weisman MH Buckner JH Gregersen PK. Omega-3 Fatty Acids are Associated With a Lower Prevalence of Autoantibodies in Shared Epitope-Positive Subjects at Risk for Rheumatoid Arthritis. Ann Rheum Dis (2017) 76(1):147–52. doi: 10.1136/annrheumdis-2016-209154 PMC5371398 27190099

[26] Sparks JA O’Reilly ÉVerifytat Barbhaiya M Tedeschi SK Malspeis S Lu B. Association of Fish Intake and Smoking With Risk of Rheumatoid Arthritis and Age of Onset: A Prospective Cohort Study. BMC Musculoskel Dis (2019) 20(1):2. doi: 10.1186/s12891-018-2381-3 PMC6320647 30611246

[27] Kallberg H Padyukov L Plenge RM Ronnelid J Gregersen PK van der Helm-van Mil AH. Gene-Gene and Gene-Environment Interactions Involving HLA-DRB1, PTPN22, and Smoking in Two Subsets of Rheumatoid Arthritis. Am J Hum Genet (2007) 80(5):867–75. doi: 10.1086/516736 PMC1852748 17436241

[28] Costenbader KH Feskanich D Mandl LA Karlson EW. Smoking Intensity, Duration, and Cessation, and the Risk of Rheumatoid Arthritis in Women. Am J Med (2006) 119(6):503.e1–9. doi: 10.1016/j.amjmed.2005.09.053 16750964

[29] Unriza-Puin S Bautista-Molano W Lafaurie GI Valle-Oñate R Chalem P Chila-Moreno L. Are Obesity, ACPAs and Periodontitis Conditions That Influence the Risk of Developing Rheumatoid Arthritis in First-Degree Relatives? Clin Rheumatol (2017) 36(4):799–806. doi: 10.1007/s10067-016-3519-z 28028684

[30] Zemedikun DT Chandan JS Raindi D Rajgor AD Gokhale KM Thomas T. Burden of Chronic Diseases Associated With Periodontal Diseases: A Retrospective Cohort Study Using UK Primary Care Data. BMJ Open (2021) 11. doi: 10.1136/bmjopen-2020-048296 PMC8689170 34924359

[31] Hansson M Falahee M Raza K. Genomic and Biological Risk Profiling: From Medicalization to Empowerment. In: Kihlbom U Hansson M Schicktanz S, editors. Ethical, Social and Psychological Impacts of Genomic Risk Communication. London: Routledge (2020).

[32] Falahee M Simons G Raza K Stack RJ. Healthcare Professionals’ Perceptions of Risk in the Context of Genetic Testing for the Prediction of Chronic Disease: A Qualitative Metasynthesis. J Risk Res (2018) 21(2):129–66. doi: 10.1080/13669877.2016.1153503

[33] van Boheemen L Turk S Beers-Tas MV Bos W Marsman D Griep EN. Atorvastatin is Unlikely to Prevent Rheumatoid Arthritis in High Risk Individuals: Results From the Prematurely Stopped STAtins to Prevent Rheumatoid Arthritis (STAPRA) Trial. RMD Open (2021) 7. doi: 10.1136/rmdopen-2021-001591 PMC7942258 33685928

[34] van Boheemen L ter Wee MM Seppen B van Schaardenburg D. How to Enhance Recruitment of Individuals at Risk of Rheumatoid Arthritis Into Trials Aimed at Prevention: Understanding the Barriers and Facilitators. RMD Open (2021) 7. doi: 10.1136/rmdopen-2021-001592 PMC7942248 33685929

[35] de Bekker-Grob E Berlin C Levitan B Raza K Christoforidi K Cleemput I. Giving Patients’ Preferences a Voice in Medical Treatment Life Cycle: The PREFER Public–Private Project. Patient: Patient Centred Outcomes Res (2017) 10(3):263–6. doi: 10.1007/s40271-017-0222-3 28247251

[36] Ho MP Gonzalez JM Lerner HP Neuland CY Whang JM McMurry-Heath M. Incorporating Patient-Preference Evidence Into Regulatory Decision Making. Surg Endosc (2015) 29(10):2984–93. doi: 10.1007/s00464-014-4044-2 25552232

[37] Bouvy J Cowie L Lovett R Morrison D Livingstone H Crabb N. Use of Patient Preference Studies in HTA Decision Making: A NICE Perspective. patient (2020) 13:145–9. doi: 10.1007/s40271-019-00408-4 31942698

[38] Ho M Saha A McCleary KK Levitan B Christopher S Zandlo K. A Framework for Incorporating Patient Preferences Regarding Benefits and Risks Into Regulatory Assessment of Medical Technologies. Value Health J Int Soc Pharmacoeconomics Outcomes Res (2016) 19(6):746–50. doi: 10.1016/j.jval.2016.02.019 27712701

[39] Marsh K van Til JA Molsen-David E Juhnke C Hawken N Oehrlein EM. Health Preference Research in Europe: A Review of Its Use in Marketing Authorization, Reimbursement, and Pricing Decisions-Report of the ISPOR Stated Preference Research Special Interest Group. Value Health J Int Soc Pharmacoeconomics Outcomes Res (2020) 23(7):831–41. doi: 10.1016/j.jval.2019.11.009 32762984

[40] Smith SG Sestak I Forster A Partridge A Side L Wolf MS. Factors Affecting Uptake and Adherence to Breast Cancer Chemoprevention: A Systematic Review and Meta-Analysis. Ann Oncol (2016) 27(4):575–90. doi: 10.1093/annonc/mdv590 PMC4803450 26646754

[41] Lemstra M Blackburn D Crawley A Fung R. Proportion and Risk Indicators of Nonadherence to Statin Therapy: A Meta-Analysis. Can J Cardiol (2012) 28(5):574–80. doi: 10.1016/j.cjca.2012.05.007 22884278

[42] Novotny F Haeny S Hudelson P Escher M Finckh A. Primary Prevention of Rheumatoid Arthritis: A Qualitative Study in a High-Risk Population. Joint Bone Spine (2013) 80(6):673–4. doi: 10.1016/j.jbspin.2013.05.005 23835304

[43] Newsum EC van der Helm-van Mil AH Kaptein AA. Views on Clinically Suspect Arthralgia: A Focus Group Study. Clin Rheumatol (2016) 35(5):1347–52. doi: 10.1007/s10067-015-3038-3 PMC4844635 26272058

[44] Stack RJ Stoffer M Englbrecht M Mosor E Falahee M Simons G. Perceptions of Risk and Predictive Testing Held by the First-Degree Relatives of Patients With Rheumatoid Arthritis in England, Austria and Germany: A Qualitative Study. BMJ Open (2016) 6(6):e010555–e. doi: 10.1136/bmjopen-2015-010555 PMC4932277 27357193

[45] Falahee M Simons G Buckley CD Hansson M Stack RJ Raza K. Patients' Perceptions of Their Relatives' Risk of Developing Rheumatoid Arthritis and of the Potential for Risk Communication, Prediction, and Modulation. Arthrit Care Res (2017) 69(10):1558–65. doi: 10.1002/acr.23179 27998043

[46] Simons G Stack RJ Stoffer-Marx M Englbrecht M Mosor E Buckley CD. Perceptions of First-Degree Relatives of Patients With Rheumatoid Arthritis About Lifestyle Modifications and Pharmacological Interventions to Reduce the Risk of Rheumatoid Arthritis Development: A Qualitative Interview Study. BMC Rheumatol (2018) 2:31. doi: 10.1186/s41927-018-0038-3 30886981 PMC6390593

[47] Munro S Spooner L Milbers K Hudson M Koehn C Harrison M. Perspectives of patients, first-degree relatives and rheumatologists on preventive treatments for rheumatoid arthritis: a qualitative analysis. BMC Rheumatol (2018) 2(1):18. doi: 10.1186/s41927-018-0026-7 30886969 PMC6390586

[48] Mosor E Stoffer-Marx M Steiner G Raza K Stack RJ Simons G. I Would Never Take Preventive Medication! Perspectives and Information Needs of People Who Underwent Predictive Tests for Rheumatoid Arthritis. Arthritis Care Res (Hoboken) (2020) 72(3):360–8. doi: 10.1002/acr.23841 PMC7064954 30710453

[49] Singhal J Wells I Simons G Wöhlke S Raza K Falahee M. Public Perceptions of Predictive Testing for Rheumatoid Arthritis Compared to Breast Cancer and Early-Onset Alzheimer’s Disease: A Qualitative Study. BMC Rheumatol (2022) 6(1):14. doi: 10.1186/s41927-021-00244-w 35232494 PMC8889636

[50] Marshall AA Zaccardelli A Yu Z Prado MG Liu X Miller Kroouze R. Effect of Communicating Personalized Rheumatoid Arthritis Risk on Concern for Developing RA: A Randomized Controlled Trial. Patient Educ Couns (2019) 102(5):976–83. doi: 10.1016/j.pec.2018.12.011 PMC6491232 30558852

[51] Finckh A Escher M Liang MH Bansback N. Preventive Treatments for Rheumatoid Arthritis: Issues Regarding Patient Preferences. Curr Rheumatol Rep (2016) 18(8):51. doi: 10.1007/s11926-016-0598-4 27402108

[52] Harrison M Spooner L Bansback N Milbers K Koehn C Shojania K. Preventing Rheumatoid Arthritis: Preferences for and Predicted Uptake of Preventive Treatments Among High Risk Individuals. PloS One (2019) 14(4):e0216075. doi: 10.1371/journal.pone.0216075 31022252 PMC6483264

[53] Harrison M Bansback N Aguiar M Koehn C Shojania K Finckh A. Preferences for Treatments to Prevent Rheumatoid Arthritis in Canada and the Influence of Shared Decision-Making. Clin Rheumatol (2020) 39(10):2931–41. doi: 10.1007/s10067-020-05072-w 32248434

[54] van Boheemen L Bolt JW ter Wee MM de Jong HM van de Sande MG van Schaardenburg D. Patients’ and Rheumatologists’ Perceptions on Preventive Intervention in Rheumatoid Arthritis and Axial Spondyloarthritis. Arthritis Res Ther (2020) 22(1):217. doi: 10.1186/s13075-020-02314-9 32933547 PMC7493385

[55] Wells I Zemedikun DT Simons G Stack RJ Mallen CD Raza K. Predictors of Interest in Predictive Testing for Rheumatoid Arthritis Amongst First Degree Relatives of Rheumatoid Arthritis Patients. Rheumatol (Oxford) (2021). doi: 10.1093/rheumatology/keab890 PMC9348622 34850849

[56] Durand C Eldoma M Marshall DA Bansback N Hazlewood GS. Patient Preferences for Disease-Modifying Antirheumatic Drug Treatment in Rheumatoid Arthritis: A Systematic Review. J Rheumatol (2020) 47(2):176–87. doi: 10.3899/jrheum.181165 30988125

[57] Simons G Caplan J DiSantostefano RL Veldwijk J Englbrecht M Bywall KS. Systematic Review of Quantitative Preference Studies of Treatments for Rheumatoid Arthritis Among Patients and at-Risk Populations. Arthritis Res Ther (2022) 24(1):55. doi: 10.1186/s13075-021-02707-4 35193653 PMC8862509

[58] Falahee M Simons G DiSantostefano RL Valor Méndez L Radawski C Englbrecht M. Treatment Preferences for Preventive Interventions for Rheumatoid Arthritis: Protocol of a Mixed Methods Case Study for the Innovative Medicines Initiative PREFER Project. BMJ Open (2021) 11. doi: 10.1136/bmjopen-2020-045851 PMC8039213 36916312

[59] Simons G Veldwijk J DI Santostefano R Englbrecht M Radawski C Valor L. OP0160-HPR Preferences For Treatments To Prevent Rheumatoid Arthritis: Discrete Choice Survey Of General Populations In United Kingdom, Germany, And Romania. Ann Rheum Dis (2021) 80:96–7. doi: 10.1136/annrheumdis-2021-eular.2168

[60] Falahee M Finckh A Raza K Harrison M. Preferences of Patients and At-Risk Individuals for Preventive Approaches to Rheumatoid Arthritis. Clin Ther (2019) 41(7):1346–54. doi: 10.1016/j.clinthera.2019.04.015 31196645

[61] Simons G Mallen CD Kumar K Stack RJ Raza K. A Qualitative Investigation of the Barriers to Help-Seeking Among Members of the Public Presented With Symptoms of New-Onset Rheumatoid Arthritis. J Rheumatol (2015) 42:585–92. doi: 10.3899/jrheum.140913 PMC4544743 25641894

[62] Simons G Mason A Falahee M Kumar K Mallen CD Raza K. Qualitative Exploration of Illness Perceptions of Rheumatoid Arthritis in the General Public. Musculoskeletal Care (2017) 15(1):13–22. doi: 10.1002/msc.1135 26833593 PMC4903170

[63] Simons G Belcher J Morton C Kumar K Falahee M Mallen CD. Symptom Recognition and Perceived Urgency of Help-Seeking for Rheumatoid Arthritis and Other Diseases in the General Public: A Mixed Method Approach. Arthrit Care Res (2017) 69(5):633–41. doi: 10.1002/acr.22979 PMC5518890 27389847

[64] Sparks JA Iversen MD Yu Z Triedman NA Prado MG Miller Kroouze R. Disclosure of Personalized Rheumatoid Arthritis Risk Using Genetics, Biomarkers, and Lifestyle Factors to Motivate Health Behavior Improvements: A Randomized Controlled Trial. Arthrit Care Res (2018) 70(6):823–33. doi: 10.1002/acr.23411 PMC5897224 29024454

[65] Russo S Monzani D Pinto CA Vergani L Marton G Falahee M. Taking Into Account Patient Preferences: A Consensus Study on the Assessment of Psychological Dimensions Within Patient Preference Studies. Patient Prefer Adherence (2021) 15:1331–45. doi: 10.2147/PPA.S261615 PMC8219660 34177261

[66] Russo S Jongerius C Faccio F Pizzoli SFM Pinto CA Veldwijk J. Understanding Patients' Preferences: A Systematic Review of Psychological Instruments Used in Patients' Preference and Decision Studies. Value Health J Int Soc Pharmacoeconomics Outcomes Res (2019) 22(4):491–501. doi: 10.1016/j.jval.2018.12.007 30975401

[67] Bywall KS Veldwijk J Hansson MG Baecklund E Raza K Falahee M. Does Being Exposed to an Educational Tool Influence Patient Preferences? The Influence of an Educational Tool on Patient Preferences Assessed by a Discrete Choice Experiment. Patient Educ Couns (2021) 104(10):2577–85. doi: 10.1016/j.pec.2021.03.013 33757643

