# Supplementary material for: Perspectives of at-Risk Individuals on Preventive Intervention for Rheumatoid Arthritis: A Mini Review
Source: Front Immunol. 2022 Apr 29;13:883287. doi: 10.3389/fimmu.2022.883287 (PMC9098966; doi:10.3389/fimmu.2022.883287)
Supplement: Supplementary file 1 [file DataSheet_1.docx]

Supplementary Material

**Search strategy used to identify articles for inclusion in the mini review**

The authors of this review have previously been authors of two systematic review studies that have addressed stakeholder preferences for predictive and preventive strategies for rheumatoid arthritis. The articles identified in a recent systematic review of related qualitative studies (1) and another of quantitative studies (2) were included in the current review. To extend and update this previous work a PubMed search was carried out (February 2022) using the following search terms:

(preference* OR perception* OR perspective* OR view*) AND "rheumatoid arthritis" AND (risk OR prevent* or predict*)

This search strategy was designed to be comprehensive since this is a nascent research area. The strategy follows the PECO framework (3): Eligible articles were full papers describing primary research addressing the following criteria:

| Population(s) | **At risk of rheumatoid arthritis (EULAR at-risk stages a-d (4)):**  Genetic/environmental risk factors (e.g., first-degree relatives)  OR  Systemic autoimmunity associated with RA (e.g., Rheumatoid Factor/ACPA* positive individuals)  OR  Symptoms without clinical synovitis (e.g., clinically suspect arthralgia) |
| --- | --- |
| Exposure(s) | **Risk assessment / predictive testing for rheumatoid arthritis**  OR  **Preventive intervention for rheumatoid arthritis** |
| Comparator | Not applicable |
| Outcomes | **Perceptions/preferences of stakeholders**, including:  At-risk groups (see ‘Population’ above)  Patients with rheumatoid arthritis  Healthcare professionals involved in the management of rheumatoid arthritis  Public |

*Anti-citrullinated protein antibodies

To provide a comprehensive overview within the scope of the journal requirements for a mini review article, no studies were excluded due to risk of bias, and relevant aspects of study design are discussed in the main body of the manuscript. However, methodological quality of the majority of studies included in the current review has been assessed in detail in the systematic review studies previously published by the authors.(1, 2)

**References:**

1. Siddle HJ, Chapman LS, Mankia K, Zăbălan C, Kouloumas M, Raza K, et al. Perceptions and experiences of individuals at-risk of rheumatoid arthritis (RA) knowing about their risk of developing RA and being offered preventive treatment: systematic review and thematic synthesis of qualitative studies. Ann Rheum Dis. 2021.

2. Simons G, Caplan J, DiSantostefano RL, Veldwijk J, Englbrecht M, Bywall KS, et al. Systematic review of quantitative preference studies of treatments for rheumatoid arthritis among patients and at-risk populations. Arthritis Res Ther. 2022;24(1):55.

3. Morgan RL, Whaley P, Thayer KA, Schünemann HJ. Identifying the PECO: A framework for formulating good questions to explore the association of environmental and other exposures with health outcomes. Environ Int. 2018;121(Pt 1):1027-31.

4. Gerlag DM, Raza K, van Baarsen LGM, Brouwer E, Buckley CD, Burmester GR, et al. EULAR recommendations for terminology and research in individuals at risk of rheumatoid arthritis: report from the Study Group for Risk Factors for Rheumatoid Arthritis. Ann Rheum Dis. 2012;71:638-41.
